# Trends in nephrology: from MANELS to a balanced representation of all community members

**DOI:** 10.1093/ndt/gfag044

**Published:** 2026-02-24

**Authors:** Lucia Del Vecchio

**Affiliations:** Department of Nephrology and Dialysis, ASST Lariana, Como, Italy

You are sitting in your seat at a medical congress attending an interesting session. The two chairs and all the speakers excepting one are males. Would you be surprised by that? Would you feel somehow uncomfortable? Does it mean that there is shortage of remarkable women in the day to day working life? Well, these questions may have very different answers, reflecting your personality, education, and culture, gender, age, ethical convincement, and religion. Apart nuances and interpretations, these types of session can be labelled precisely with a name: MANELS.

MANEL is the acronymous made by the union of male + panel. It stands for a panel of speakers composed entirely of men, typically at conferences, seminars, industry events, or in media discussions (Fig. 1). A similar wording is MANFERENCE; its meaning is obvious.

**Figure 1:**
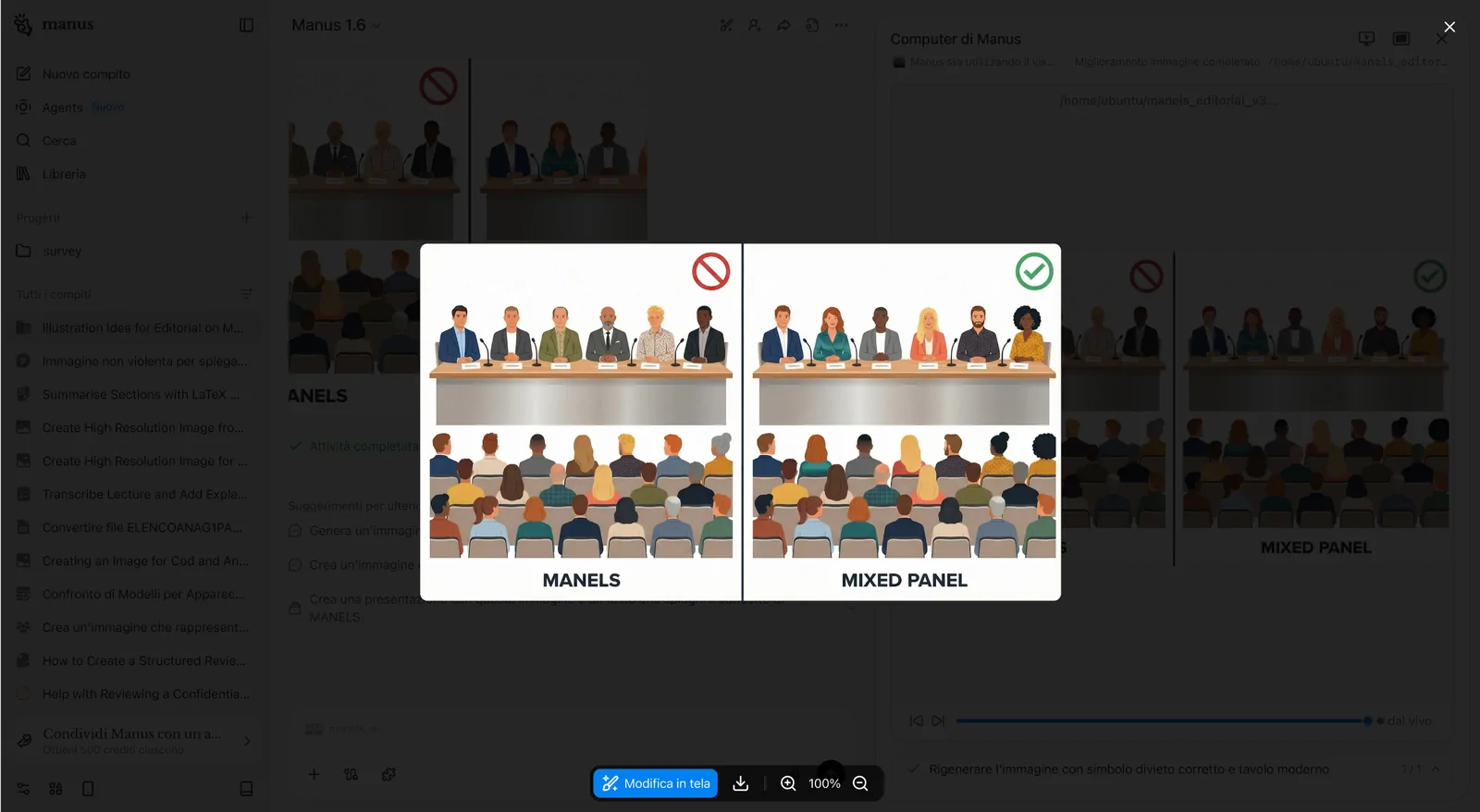
Comparative illustration of gender composition in panels. This figure provides a visual guide to best practices for gender diversity in professional and academic panels. The left side depicts a ‘manel’—a panel composed entirely of men—indicated as a non-preferred format. The right side shows a gender-balanced panel, with an equal number of male and female participants, indicated as the preferred format. This illustration was generated by the artificial intelligence platform Manus, under the direct guidance and input of the author, to visually articulate the concept.

While the term is well known in the setting of Diversity, Equity, and Inclusion, it is still little used in wider contexts and unfortunately still widely considered as acceptable. According to a survey conducted by the Women’s Inclusive Network, 75% of respondents reported that they would still attend a MANEL session, although about two–thirds would do so ‘reluctantly’; only 11% stated they would not [1]. Increasingly, MANELS have become the symbol of an old-fashion and inequitable model of the society, where single individuals are denied opportunities they deserve only because of their gender, race, or other specific characteristics independently from their true value and expertise.

In the field of medicine this is even more disturbing, since the prevalence of females has been progressively increasing in recent years, being 45% in Europe in 2010 and 53% in 2022 [2]. This shows a steady ‘feminization’ of the medical workforce. Looking to nephrology, according to data from the European Renal Association (ERA), in 2023, 52% of the members were females, with the highest representation in the younger age groups.

Despite improvements in the awareness of the importance to improve and abolish gender gap, the high female representation has not translated yet in equitable professional opportunities and achievements in the work setting.

## WHY MANELS ARE A PROBLEM?

Looking superficially to MANELS, this could be considered a non-problem: males are simply invited or included more often in medical conferences or panels simply because they are more expert than women in each topic, or because they have invested more in their career and thus obtained more recognition for the obtained results. This could be partly true for older age groups where male doctors are more prevalent; accordingly, they have received more opportunities and, at a time of career recognition, they have a higher likelihood than females to have become a recognized expert in a specific field.

But it is not as simple as that. The uneven distribution of expertise is often the consequence of unequal opportunities to climb the professional ladder, especially in younger age groups where gender distribution is balanced. This inappropriately low female representation in professional life and scientific contexts has several hidden consequences. First, it causes a lack of representation of the female contribution, with the loss of different perspective and angles. Excluding gender diversity results in a narrow scope of discussion, which can affect the quality and inclusivity of the insights presented. This is testified by the fact that in scientific medical literature mixed–gender teams produce more novel and highly cited work than same–gender teams, implying that homogenous groups (including all–male ones) underperform on innovation and impact [3]. Furthermore, panel composition shapes priorities in perspectives, clinical experiences, and research leading to a man-driven research strategy. Also, when men dominate visible roles, women engage less in public discussion, which can further harm reputation-building and networking [4]. MANELS also represent the unfair signal that male perspective and presence are more valued or authoritative, reinforcing the existing status quo even in fields, as medicine, where women account for a large share of the workforce and scientific output.

When men dominate visible expert roles, they become the default names that organizers recall, making future invitations more likely to go to the same circle, while women remain less known and thus less likely to be suggested.

Finally, when women are systematically under–invited, the few available positions become symbolic ‘tokens’ that can set women in competition with one another and undermine collective advocacy for fairer representation. By contrast, routinely achieving a more balanced gender distribution on panels and committees broadens the sense of opportunity, fostering collaboration, mutual sponsorship, and a shared willingness to challenge the norms that sustain manels.

## MANELS IN MEDICINE AND NEPHROLOGY: FROM ACCEPTED PRACTICE TO ACTION

Back in 2019, *The Lancet* published a pioneering editorial addressing the issue of MANELS in medicine [5]. In this editorial, the Lancet Group clearly stated its commitment to combat gender underrepresentation. Specifically, they pledged not to serve as panellists at public conferences or events featuring all-male panels. They also committed to ensuring at least 50% female representation among speakers at events they organize or plan, inviting women not only as chairs or moderators but also as panellists.

But this is not the only initiative. Years ago, the American Society of Clinical Oncology decided to instruct avoid MANELS except in extraordinary situations. Following that, the percentage of MANELS at the American Annual Meeting decreased from 17.4% in 2018 to 9.9% in 2021 [6]. While the percentage of women oncologists invited as panellists progressively increased, they remained underrepresented for the topics of genitourinary cancers, suggesting a higher gender gap in surgical specialties translating into a higher burden of MANELS. Accordingly, when looking at data from urological meetings, more than half of the sessions are still held by MANELS [7].

The American Society of Nephrology (ASN) has been the first to raise awareness of gender gap in the specialty by creating the association Women in Nephrology (WIN) >30 years ago. This has translated in several efforts and initiatives, including actions to increase the percentage of women invited at the annual Kidney Week, leadership programmes, and the creation of a speaker bureau to diversify panels and invited talks. Specifically, this is an online directory of WIN members who have explicitly volunteered to be contacted as speakers, panellists, or collaborators, with listed areas of expertise. The bureau is open to all the genders. The efforts have translated into a significant increase in the percentage of women moderators and speakers [8]. However, women continued to be under–represented among early programme chairs and major award recipients [8].

Like ASN, the ERA has actively advanced initiatives to promote gender equity and broader diversity within the nephrology community. ERA now explicitly requests fair geographic and gender representation when constituting its internal commissions and working groups. The same holds true for the invitations of chairs and speakers at the ERA annual meetings. In 2025, on 1365 speakers at the ERA Congress in Vienna, 51% were women, indicating a successful achievement of gender balance. When looking at the winners of ERA awards, in 2025 there was still a slightly male predominance (7 over 12, 58%). This represents a remarkable improvement, considering that the female proportion was only 23% 10 years before [9]. However, the 2025 industry–sponsored symposia painted a different picture: only 26% of chairs and 41% of speakers were women. This pattern implies that gender–equity barriers remain substantially higher in industry–driven events than within the ERA–led scientific programme, especially outside the ERA umbrella. This is notable, given that large multinational pharmaceutical companies generally have internal policies and guidelines to support diversity and inclusion.

## IMPOSTOR PHENOMENON AND STRUCTURAL BARRIERS IN NEPHROLOGY

For a long time, MANELS were implicitly treated as natural and even justified, on the assumption that women lacked either the necessary competence or the ‘right’ disposition to advance to senior positions and be recognized as experts. These assumptions became self–fulfilling. If women were not seen as potential leaders, they were less likely to be mentored, sponsored, or nominated for visible roles, which in turn meant fewer women with the titles and profiles organizers look for when selecting speakers. Work–life integration pressures (pregnancy, caregiving, unequal domestic responsibilities) further compress the time and energy available for networking, travel to conferences, and unpaid committee work that often serves as the informal currency of advancement.

On top of this, many women report impostor feelings—persistent self-doubt and fear of not being ‘good enough’—which can make them more hesitant to put themselves forward for competitive grants, plenaries, or high-profile panels [10].

In 2025, the Women of ERA (WERA) Task Force developed a survey to examine gender disparities among the ERA members. This was a validated questionnaire covering six domains: professional background, workplace environment, leadership, work–life balance, discrimination, and impostor syndrome (IPSS-3 scale) [11]. According to preliminary data, women suffered more than men of impostor syndrome at all age groups. When looking at the likelihood of being a congress speaker, men report more often to have given more than five times a presentation at national or international congresses (54.2% vs 36%).

Impostor phenomenon could be a reason making women more likely to decline invitations, especially when asked to speak outside their narrow comfort zone or in highly visible plenary settings, while men may be more willing to accept such invitations. According to the WERA survey, women score higher for impostor syndrome than men (60.2% vs 50.9% of the maximum IPSS-3 scale; *P* < .001). Notably, being a frequent speaker is protective over impostor syndrome for both genders. However, women still score higher than men also in this favourable context (unpublished data).

## HOW TO MOVE BEYOND MANELS IN NEPHROLOGY

MANELS must be relegated to the past. Moving forward requires comprehensive, multifaceted solutions. Ensuring that women are chosen as invited or featured speaker at society conferences is just the first step. Such invitations offer a high-status platform that can raise the profile of their research and careers. Moreover, studies show a clear positive association between the number of women involved in organizing a symposium and the proportion of women who appear on its speaker list [12]. In this respect, both ASN and ERA have become virtuous realities. However, the final goal is not to increase the number of female speakers only because they are women, but to make them felt as true experts as men. Achieving this is far more complex, as it requires dismantling the structural barriers that prevent women from advancing professionally and gaining recognition.

Inside this commitment, in 2024 ERA created the WERA Task Force aimed at achieving gender balance within the field of nephrology by encouraging female nephrologists to actively contribute to scientific research, career development, and dissemination, particularly by increasing their participation as speakers at educational events. The WERA Task Force has also invested in concrete capacity–building initiatives, including supporting several women nephrologists to attend leadership courses at the University of Oxford. The scholarships were supported by the ERA Curatorium with contributions from industry partners, signalling that pharmaceutical companies are increasingly willing to invest concretely in efforts to improve gender balance in nephrology. The expertise gained through these programmes is now being channelled into structured one–to–one mentorship for early–career women nephrologists, with the explicit aim of fostering engagement with the profession, strengthening self–confidence, and nurturing a new generation of recognized experts and leaders in nephrology.

In parallel, it is essential to engage pharmaceutical companies, which sponsor a substantial proportion of congress symposia and advisory boards, to extend their public commitments to explicit avoid MANELS in industry–sponsored symposia, satellite sessions, and advisory boards inside and outside the ERA reality. Only if both scientific societies and industry partners align on these standards it will be possible to avoid ‘two parallel worlds’ in which the main programme progresses towards parity while sponsored content continues to lag behind.
